# MXene-Based High-Performance Soft Pressure Sensor Using Gel–Deep Eutectic Solvent Composite

**DOI:** 10.3390/mi16050579

**Published:** 2025-05-15

**Authors:** Riku Sasaki, Kaiin Tou, Shoma Kamanoi, Junya Yoshida, Yoshihito Takabe, Yasuyuki Miura, Eri Kamiya, Ayana Hirayama, Tomohito Sekine

**Affiliations:** Graduate School of Organic Materials Science, Yamagata University, 4-3-16, Jonan, Yonezawa 992-8510, Yamagata, Japan

**Keywords:** MXene, pressure sensor, composite material, deep eutectic solvent, wearable electronics

## Abstract

MXene, a layered nanocarbon material, exhibits excellent conductivity and solubility. Its high sensitivity also makes it useful for soft pressure sensors. However, the compatibility between sensitivity and fast responses in resistance-change sensors remains a major issue. This study developed an MXene-based high-performance soft pressure sensor using a gel–deep eutectic solvent composite. The composite conductive material exhibited excellent solubility and printability in soft device fabrication. The aim of this work was to produce a high-quality soft pressure sensor that exhibited quick responses over a wide sensitivity range for detecting applied pressure. The sensors achieved high performance in terms of a high-speed response (40 ms) and good sensitivity (−0.0109 kPa^−1^). These results represent an advance in intelligent wearable sensing systems by combining materials science and electronic devices.

## 1. Introduction

MXene refers to a two-dimensional (2D) conductive material comprising layers of atoms bonded by van der Waals forces [1,2,3] with transition metals between the carbon layers. Such 2D materials have attracted wide scientific interest because of their unique chemical, physical, and electrical properties. When forming thin films, their useful properties are expected to be alterable by changing the transition metal species [4,5,6], making such 2D materials suitable for a variety of applications, including sensors, energy storage, and photonic devices [7,8,9]. In addition, their excellent solubility makes them suitable for printing processes [10,11,12]. For this reason, 2D materials are being studied widely, from the analysis of chemical properties in the field of materials science to the fabrication of high-performance electronics and devices.

Flexible sensors have attracted significant interest among materials scientists and electronic engineers since the 2000s. More recently, interest in flexible sensors has expanded to include the biomedical, robotics, and Artificial Intelligence (AI) domains [13,14,15]. In particular, physical sensors, either embedded or wearable, that can detect phenomena in the outside world have been developed in great numbers. Among them, pressure sensors fabricated from nanocarbon and 2D materials exhibit attractive physical properties such as high ion transport, excellent thin-film technology in combination with surfactants, and high electrical conductivity. Research has been actively conducted to improve the sensitivity of these sensors, expand their dynamic range, and develop new electronics, such as wearable devices [16,17,18].

However, achieving both high sensitivity and high-speed detection remains a challenge for thin-film pressure sensors using MXenes. The sensitivity of these resistance-change sensors is calculated from the decay rate of the initial resistance due to the applied pressure [19,20]. In general, thicker films exhibit better sensor characteristics because a greater initial resistance results in poorer deformation sensitivity during pressure sensing and mechanical noise. This initial resistance is inversely proportional to the film’s thickness; therefore, the thinner the film, the lower the initial resistance [21]. However, the more easily implemented thinner films are desirable for wearable devices [22]. In addition, resistance-change sensors operate on the principle that the resistance value changes owing to changes in the physical contact of a conductor caused by pressure. Therefore, their response to pressure tends to be slower than that of other sensors (capacitive and piezoelectric types) [23].

Wearable, flexible, and soft physical sensors utilizing MXene have reportedly achieved high sensitivity based on their exceptional electrical conductivity [24]. Furthermore, by leveraging its outstanding flexibility, MXene has been applied to health- and oral-care devices. These advancements represent significant progress aimed at integrating this material into biometric information management and similar systems [25]. Furthermore, high sensitivity and a fast response time are important factors in force control when considering applications such as healthcare and robot e-skin; therefore, improving performance is desirable. Because thinness is important for implementation, ensuring a thin, high-sensitivity film with short response times using MXenes is essential for developing attractive, high-performance sensors. Specifically, pressure sensors designed to quantify tactile sensations during object gripping require both high sensitivity and rapid response performance. From the perspective of materials science, these properties can be achieved through the construction of material systems, particularly by utilizing innovative combinations of MXene and surfactants. Utilizing deep eutectic solvents (DESs) as surfactants is particularly effective in preventing the aggregation of composite inks, enabling the formation of a highly uniform pressure-sensitive layer [26]. Previous studies have highlighted remarkable advancements in composite materials, including the expanded dynamic range achieved through MXene–polymer composites [27]. This research specifically focuses on film-forming properties and demonstrates the feasibility of creating printable pressure sensors by employing DES-based ink formulations. Moreover, MXene exhibits high electrical conductivity and demonstrates lower aggregation within composite materials compared to other nanocarbon materials [2]. This property enables the fabrication of high-performance thin-film sensors using printing- or solution-based methods. This characteristic is one of the key advantages of the MXene–DES combination.

This study achieved both high sensitivity and fast responsiveness in a thin-film pressure sensor using a completely new material system that combines MXene, polyvinyl alcohol (PVA) gel, and a new eutectic solvent in the pressure-sensitive layer. We developed a functional conductive solvent by mixing appropriate amounts of MXene, which uses titanium as the transition metal, PVA gel that can soften the sensor housing, and a new eutectic solvent to change the crystal orientation of MXene and enhance conductivity. The resulting printed pressure sensor was a thin film <1 mm thick that exhibited a high-speed response (40 ms) and good sensitivity (−0.0109 kPa^−1^). This was due to the new eutectic solvent, which enabled the MXene crystals to align isotopically. Furthermore, this pressure sensor was applied to a wearable device and used in a force-detection system for grasping an object. This efficient, theoretically developed wearable e-skin sensor was experimentally demonstrated to be feasible for deployment in a real-time tactile sensing system.

## 2. Materials and Methods

First, a 300 nm thick mixture of polyvinylphenol (PVP) and melamine resin was spin-coated onto a Polyethylene Naphthalate (PEN) substrate (100 nm) as a smoothing layer and was then annealed at 150 °C for 1 h. Poly(2,3-dihydrothieno-1,4-dioxin)-poly(styrenesulfonate) (PEDOT:PSS) (SV4-STAB, Heraeus, Hanau, Germany) was stencil-printed on top of the smoothing layer and annealed at 120 °C for 30 min [28]. Subsequently, a composite solution of PVA, MXene (Ti_3_C_2_, Japan Material Technologies Co., Tokyo, Japan), and a DES (urea and choline chloride mixed in a mass ratio of 1:1) (PVA: 37 wt%, MXene: 40 wt%, DES: 23 wt%) was deposited as a pressure-sensitive layer. In this study, Mxene (Ti_3_C_2_) was selected for its superior electrical conductivity and good dispersibility in several solvents, enabling uniform coating. These properties allow for high-performance flexible device applications utilizing coating techniques. The resulting solution was stirred at room temperature for 1 h using a magnetic stirrer [29]. The viscosity (approximately 515 mPa·s) of the obtained solution was measured using a fabricated viscometer. Further details are provided in Figure S1. This solution was poured into a mold and subjected to five cycles of freezing and thawing (−20 °C for 2 h and 4 °C for 2 h) to gel, after which it was removed from the mold to create a pressure sensor [30]. This allowed the water in the PVA to evaporate at a slow rate, creating an elastic gel. In addition, the electrostatic binding of the urea and choline chloride of the DES to the MXene surface facilitated the isotropic alignment of the crystal orientation of MXene, improving its electrical conductivity. Moreover, choline chloride has a low electrical conductivity, which reduced the initial resistance of the pressure-sensitive layer and improved its performance [31]. Next, Polydimethylsiloxane (PDMS) was spin-coated onto the substrate to form a sealing layer approximately 100 nm thick. A compression tester was used to apply pressure perpendicular to the pressure-sensitive layer, and electrical characteristics, such as sensitivity and response time, were calculated by reading the change in resistance [32]. As a resistance-change sensor, when pressure was applied to the pressure-sensitive layer, its electrical conductivity changed, causing a change in resistance. When the resistance change was detected, the device functioned as a pressure sensor. This could be modeled as a variable resistor, as depicted in Figure S2. The PEN substrate used in this study was attached to a glass substrate (support) using double-sided tape during device fabrication (JE1212C-V, Joyo Engineering, Tokyo, Japan). After fabrication, the PEN substrate was peeled off the glass substrate without affecting the device’s characteristics.

## 3. Results

**Figure 1:**
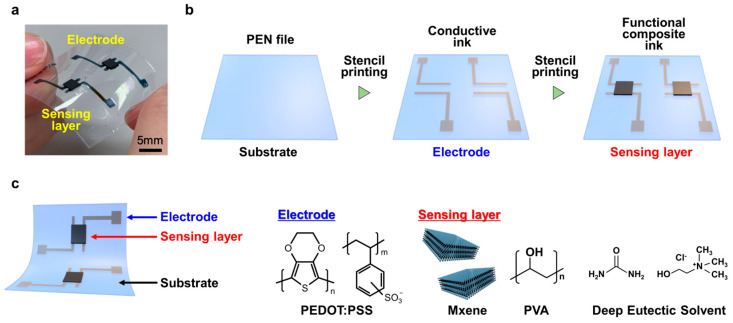
Overview of the fabricated soft pressure sensor. (**a**) External view of the sensor. The pressure-sensitive layer is deposited on the counter electrode. The distance between the counter electrodes is 1 mm. (**b**) Schematic of stencil printing fabrication method. (**c**) Cross-sectional structure of the fabricated sensor and molecular structure of the materials. The initial resistance of the pressure-sensitive layer ≈ 100 kΩ, and the electrodes’ conductivity ≈ 100 S cm^−1^.

Figure 1 contains an overview of the fabricated soft pressure sensors, with Figure 1a showing a photograph of the sensor. It is highly flexible, with a pressure-sensitive layer formed on the opposing electrode. The opposing electrodes were spaced 1 mm apart. The entire sensor was print-fabricated, and with appropriate material selection and processing, it exhibited high functionality as a soft electronic device. Figure 1b shows a schematic of the print fabrication method. The electrode and pressure-sensitive layers were deposited on the substrate in that order. This study employed stencil printing, and all electrodes and pressure-sensitive layers were fabricated using this method. Figure 1c shows the cross-sectional structure of the fabricated sensor and the molecular structure of the materials used. MXene was used as the conductive material in the pressure-sensitive layer, PVA gel served as the pressure-sensitive layer housing, and the DES was the crystallization accelerator and conductivity aid. Generally, electricity flows through MXenes when they make physical contact with each other, but our research concept was to improve the sensitivity of the sensor by aligning the crystal orientation of MXene to allow electricity to flow more efficiently and lower the initial resistance. The initial resistance of the fabricated pressure-sensitive layer was approximately 100 kΩ. The electrodes’ conductivity was approximately 100 S cm^−1^, sufficiently higher than that of the pressure-sensitive layer; thus, even a thin film fabricated by printing can function as an electrode. Previous studies have shown that the initial resistance decreases with sensitivity when pressure is below this level [19,33].

**Figure 2:**
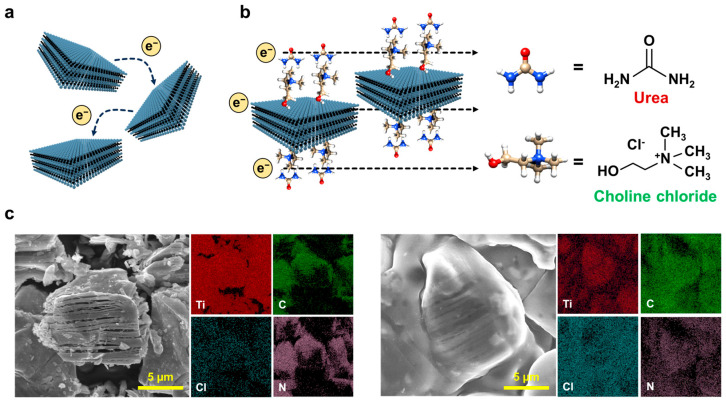
Schematic of the material system for a high-performance soft pressure sensor. (**a**) Schematic of the principle of conductivity of MXene in a typical gel; MXene randomly coordinated in a PVA gel functions as a conductive material mainly through electrical conductivity between the surface and the interior. (**b**) Conductivity principle of MXene in the functional composite material developed in this study (MXene, PVA, DES); MXene is coordinated relatively isotropically in the PVA gel, resulting in efficient conductivity. Choline chloride itself is also conductive; hence, it is expected to function as a conductive auxiliary agent. (**c**) SEM and EDS images of MXene powder only and functional composites (left: MXene powder, right: composite material). The surface morphology is very different owing to the PVA gel coating, but the presence of MXene is clearly visible. Four signals (Ti, C, Cl, and N) were detected in EDS.

Figure 2 shows a schematic of the original material system used in this study. Our sensor was a highly sensitive device because of the enhanced conductivity arising from the autonomous crystallographic alignment in the MXene in the pressure-sensitive layer. Figure 2a shows a schematic of the MXene conductivity principle, which is randomly coordinated in PVA gels and functions mainly to conduct electricity between the surface and interior. Because MXene is randomly present in a gel, its conductivity is relatively low. This is because there are obstacles to physical contact when electricity passes through the surface or interior of the randomly existing MXene. However, the functional composites developed in this study (MXene, PVA, and DES) were expected to improve conductivity because of the relatively isotropic coordination of MXene. Choline chloride is also conductive; therefore, it was expected to function as a conductive auxiliary agent (Figure 2b). Figure 2c shows the scanning electron microscope (SEM) and EDS images of MXene powder only and the functional composite materials (left: MXene powder, right: composite material). Although the surface morphology was very different because of the PVA gel coating, the presence of MXene was clearly visible. In EDS, the Ti and C signals were mainly derived from MXene, and there was no significant difference between them. In contrast, the Cl signal from choline chloride and the N signal from urea were more intense and spread throughout the membrane in the EDS images of the functional materials. This indicated that the materials were bound relatively uniformly to the MXene surfaces. These results indicated that the PVA gel and DES were uniformly and efficiently dispersed within the pressure-sensitive layer. This enabled the development of a high-performance soft pressure rapid-response sensor with high sensitivity using a thin pressure-sensitive layer, which mainly contributed to the construction of the material system.

**Figure 3:**
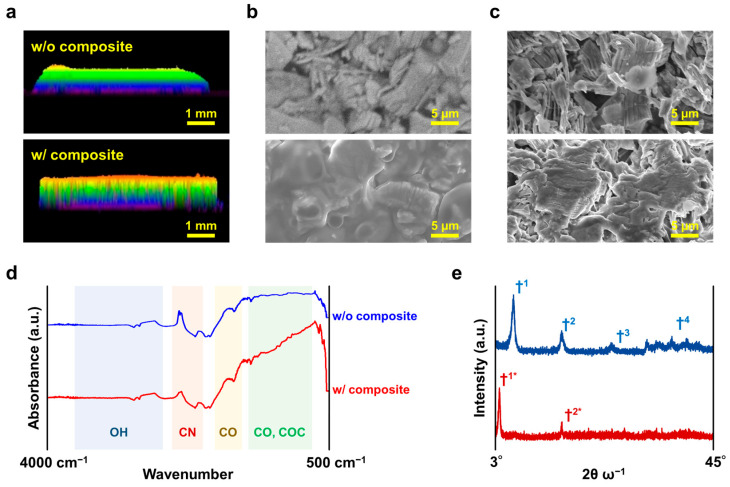
Results of the physicochemical analysis of the pressure-sensitive layers prepared. (**a**–**c**) all show the top one containing MXene only (w/o composite) and the bottom one containing composite materials (w/composite). The thickness of both pressure-sensitive layers is about 1 mm. (**a**) Cross-sectional laser microscopic images of pressure-sensitive layers prepared using PVA gel with MXene only or with composite materials, respectively. (**b**) Surface SEM image and (**c**) cross-sectional SEM image of the pressure-sensitive layer. (**d**) The results of the bonding state analysis of the compounds in the pressure-sensitive layer by FT-IR. This FT-IR was measured from 4000 to 500 cm^−1^. (**e**) XRD spectra of the respective pressure-sensitive layers are shown. This XRD was measured from 3° to 45°. The blue line represents the spectrum w/o composite and the red line represents the spectrum w/ composite. In w/o composite, peaks were detected at †^1^ through †^4^. When w/ composite was present only peaks †^1*^ and †^2*^ were observed.

Figure 3 shows the results of the physicochemical analyses of the fabricated pressure-sensitive layers, with a particular focus on the film properties. Figure 3a shows cross-sectional laser microscopy images of the pressure-sensitive layers prepared from PVA gels containing only MXene or composite materials. The top image shows only MXene, and the bottom image shows the composite material. Both films were deposited via stencil printing to a thickness of approximately 1 mm. The cross-sectional profiles did not change significantly, indicating that the composite material did not affect the macroscopic morphology of the pressure-sensitive layers. Figure 3b shows the surface SEM images of these pressure-sensitive layers. When the PVA gel contained only MXene, MXene was physically randomly assembled. In contrast, in the composites, MXene was coated with PVA gel and other materials. This may have been due to the binding of the DES to the MXene surface, which changed the PVA coverage. Furthermore, a cross-sectional SEM image of the pressure-sensitive layer was obtained (Figure 3c). Physical phenomena, such as the dispersion and morphology of MXene, were the same as those on the surface. In other words, the conductivity of the MXene in the gel was assumed to be isotropic with respect to the film thickness. To analyze the dispersion state and other details of these MXenes, physicochemical analyses using Fourier transform infrared spectroscopy (FT-IR) and X-ray diffraction (XRD) were performed. Figure 3d shows the results of the bonding state analysis of the compounds in the pressure-sensitive layer using FT-IR. FT-IR was measured from 4000 to 500 cm^−1^, where hydrogen bonds were expected between MXene and the surfactant, and CO derived from nanocarbons of MXene was detected. More detailed information regarding the morphology of the pressure-sensitive layer is given in Figure S3. Figure 3e shows the XRD patterns of each pressure-sensitive layer. The XRD pattern was measured in the range of 3° to 45°. For the MXene, numerous peaks derived from common polycrystalline materials were detected. This indicated that MXene was randomly coordinated in the PVA. Conversely, in the case of our composite, the number of peaks decreased while the †1 peak shifted toward the lower angle. The observed peak position shift was attributed to the rearrangement of Mxene within the gel matrix. Moreover, the oxidation had a minimal impact on the device properties and was deemed to be highly constrained. This phenomenon can be attributed to the encapsulation of MXene within a gel, which effectively inhibits surface oxidation [34]. This indicated that the crystal orientation of MXene, which was randomly coordinated, was aligned in the same direction to some extent, which supported the model diagram of MXene in the composite materials, as shown in Figure 2.

**Figure 4:**
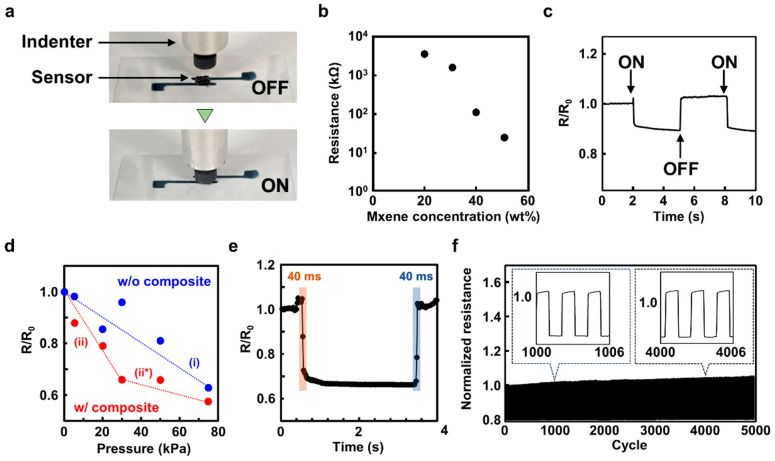
Measured electrical characteristics of the fabricated sensor. (**a**) Schematic diagram using a compression tester to apply vertical stress to the sensor. Pressure was applied by pressing the indenter perpendicularly to the pressure-sensitive layer. (**b**) Relationship between the MXene content in PVA and the initial resistance of the layer. In this study, the optimal concentration was 40 wt% MXene, which was able to optimize both the initial resistance and the solution viscosity, which was important during print deposition. (**c**) Resistance change results in a response to applied pressure. The initial resistance was R_0_, and the rate of decay of resistance R when pressure was applied was defined as R/R_0_. (**d**) Relationship of R/R_0_ and applied pressure, tested on sensors with pressure-sensitive layers containing only MXene or composite materials in PVA. The slope of each plot is denoted as (i) for the non-composite case and (ii) and (ii*) for the composite cases. (**e**) Response speed to applied pressure. The response time was about 40 ms both when pressure was applied and when pressure was released. (**f**) Cycle test results when pressure was continuously applied to the sensor. The inset graphs show representative signal data at 1000 and 4000 cycles.

Figure 4 shows the measured electrical characteristics of the fabricated sensor and schematically represents the vertical stress applied to the sensor using a compression tester. The indenter applied perpendicular pressure to the pressure-sensitive layer at a speed of 100 mm s^−1^. Figure 4b shows the relationship between the MXene content in PVA and the initial resistance of the layer. Based on the general theory of percolation concentration, we measured the concentration at which the initial resistance decreased steeply with increasing MXene content. For the sensor design, 40 wt% MXene, which optimized both the initial resistance and solution viscosity, was selected as the optimal concentration. Figure 4c shows the change in resistance in response to the applied pressure. The initial resistance was R_0_, and the decay coefficient of resistance R when pressure was applied was defined as R/R_0_. The applied pressure was approximately 5 kPa. The clear sensor signal was detected for the applied pressure. Figure 4d shows the relationship between R/R_0_ and applied pressure, which is generally linear over a relatively wide dynamic range of 5 to 75 kPa. For comparison, the sensing properties of a sensor containing only MXene in PVA gel are also shown; however, this sensor had a lower sensitivity than the composite sensor. This was attributed to the random coordination of the MXene in the PVA gel. The slope of each plot represents the sensitivity of the sensor. (i) corresponded to the non-composite sensor, whereas (ii) and (ii*) represented the composite sensors. The respective slopes were determined as follows: (i) −0.00446 kPa^−1^, (ii) −0.0109 kPa^−1^, and (ii*) −0.00189 kPa^−1^. Notably, our sensor demonstrated high sensitivity in the low-pressure range, with these slope values placing it among the high-performance flexible sensors. Furthermore, a comparative analysis with previous studies indicated that our sensor exhibited competitive performance [35]. In addition, we have included the raw data used to construct Figure 4d in Figure S4. Figure 4e shows the response speed versus the applied pressure. The pressure was set to 30 kPa. The response time was approximately 40 ms when pressure was applied and released. This was because of the elasticity of the PVA gel resulting from the freezing and thawing processes, which resulted in a high response time during sensor fabrication. In our sensor, the response time evidently depended on the duration of pressure. In Figure S5, we have documented data on the response times under different pressure application rates. Figure 4f shows the results of the cycle test when pressure was continuously applied to the sensor. The applied pressure was approximately 20 kPa, and the sensor exhibited high mechanical stability for up to 5000 cycles. The initial resistance of pressure-sensitive layers with different sizes and thicknesses is presented in Figures S6 and S7. Moreover, the physical deformation of the pressure-sensitive layer after cyclic testing is presented in Figure S8. No structural hysteresis was observed in the pressure-sensitive layer, demonstrating its high durability. Electrical hysteresis observed during the application and release of pressure on the device was also crucial for performance evaluation. Figure S9 presents data related to the hysteresis characteristics of our sensor.

**Figure 5:**
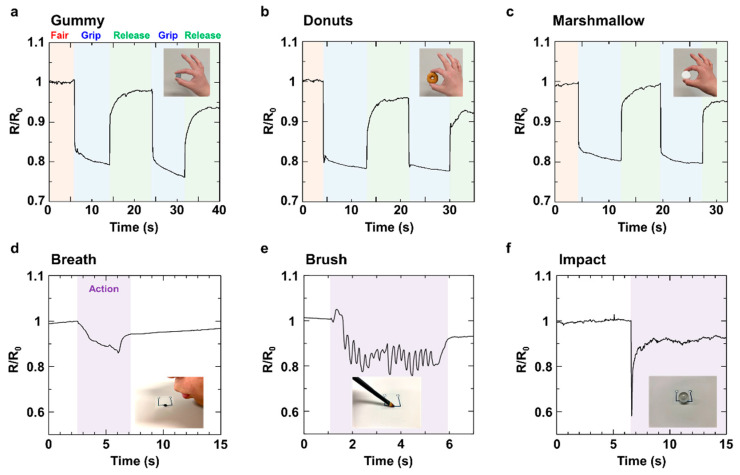
(**a**–**c**) Signal detection results of the real-time grasp test. Signals were detected by the sensor for actions such as grasping and releasing the object. The objects to be handled were gummies, doughnuts, and marshmallows. Each was gripped and released twice. (**d**–**f**) Pressure measurements in various contact scenarios. The scenarios included exhaling breath onto the surface, brushing it, and vertically dropping a marble.

Figure 5 shows the device applications of the fabricated soft pressure sensor. The sensor was mounted on a human finger, and the tactile signal was detected in real time when the finger grasped an object in Figure 5a–c. Figure 5d–f present the measurements of contact pressure under various conditions. Initially, in Figure 5a–c, our device was attached to the index finger to measure real-time signals while grasping soft objects such as gummy candy, doughnuts, and marshmallows. Clear signals were obtained for each object. Based on the maximum and minimum resistance change rates during the first grip, the grasping pressures for each object were determined as follows: gummy candy, 20 kPa; doughnut, 20 kPa; and marshmallow, 18 kPa. Furthermore, in Figure 5d–f, we measured the pressure exerted by breath, brush rubbing, and the impact of a falling marble. The marble was dropped vertically from a height of approximately 30 cm. Clear signals were successfully obtained for all cases. Based on the maximum and minimum resistance change rates, the measured pressures were as follows: breath, 5 kPa; brush rubbing, 20 kPa; and marble impact, 70 kPa. These results demonstrate that our sensor is not only applicable for wearable uses but also capable of detecting contact and impact, highlighting its potential for a wide range of applications. In Figure S10, we have included additional results from the real-time grasp test conducted in a wearable state, utilizing different fingers. Additionally, the results of the sensing characteristics obtained using weight masses are presented in Figure S11**,** which demonstrates the sensor‘s broader sensitivity to vertically applied pressure. Based on the above results, we have successfully demonstrated the extensive device applications of our sensor.

## 4. Discussion

The pressure-sensitive layer fabricated in this study is an entirely novel, thin, light, highly sensitive, and rapid-response pressure sensor based on an original material system. Various material systems using MXene have previously been proposed for pressure sensors, and many high-performance devices have been reported to exhibit improved sensitivity. However, this study is based on a novel sensor device that guarantees a high-speed response. These two physical properties will contribute to improving the functionality of future wearable tactile sensors. Many wearable devices have been used in e-skin applications in recent years, and the development of electronics with tactile functions that match those of humans is a major trend. In, e.g., robotic applications, this makes it possible to identify the grasped object instantly and move on to the next appropriate grasping motion [36,37]. The high-performance sensor developed in this study can realize high-speed movements in robots and can potentially be combined with AI and other technologies to equip intelligent and autonomous robots [38]. As a future challenge, notably, the pressure-sensitive layer, being a gel, is susceptible to changes in resistance due to water evaporation. Further optimization of the materials will allow more stable devices to be fabricated in the future.

## 5. Conclusions

This study developed an MXene-based high-performance soft pressure sensor using a gel–deep eutectic solvent composite, in which PVA gel contained optimal amounts of MXene as a conductor and a DES as a surfactant. The optimum amount of MXene was determined based on the percolation concentration theory. All the layers of this sensor were formed on a flexible substrate by the stencil printing method, making the device highly flexible and esthetically attractive. The device exhibited a linear response in terms of the resistance change to vertically applied pressure. Additionally, the device exhibited a fast response time for a resistance-change-type pressure sensor. In addition, the mechanical durability of the device was sufficient for practical applications. The force signal for grasping an object was detected in real time when the sensor was mounted on a human finger. The fact that the sensor was able to detect signals clearly, even for objects of different sizes and hardnesses, demonstrates its excellent adaptability to wearable electronics. In recent years, several wearable devices have been used in e-skin applications, and a major trend is the development of electronics with tactile functions matching those of humans. This enables, e.g., robotic applications to identify the grasped object instantly and proceed to the next appropriate grasping motion. This high-performance sensor can enable high-speed robot movements and, when integrated with AI and other technologies, will contribute to effectuating intelligent and autonomous robots.

## Data Availability

The data presented in this study are available on request from the corresponding author (Data are not publicly available due to privacy or ethical restrictions.).

## Supplementary Materials

The following supporting information can be downloaded at: https://www.mdpi.com/article/10.3390/mi16050579/s1, Figure S1: Viscosity measurement of composite ink using a viscometer.; Figure S2: The equivalent circuit model of our sensor.; Figure S3: Morphological analysis of the pressure-sensitive layer surface using an optical microscope.; Figure S4: Representative raw data used to construct Figure 4d in the main manuscript.; Figure S5: Response time when pressure is applied at different speeds.; Figure S6: Relationship between the sensing layer sizes and initial resistances.; Figure S7: Relationship between sensing layer thicknesses and initial resistances.; Figure S8: Cross-sectional images of the pressure-sensitive layer after 5000 testing cycles.; Figure S9: Signal waveform used to obtain the hysteresis graph.; Figure S10: Additional results from the real-time grasp test conducted with the wearable device, utilizing different fingers.; Figure S11: Vertical-pressure test for our sensor using weights of 10, 20, and 50 g.

## Abbreviations

DES	Deep Eutectic Solvent
FT-IR	Fourier Transform Infrared Spectroscopy
PEN	Polyethylene Naphthalate
PDMS	Polydimethylsiloxane
PVA	Polyvinyl Alcohol
PVP	Polyvinylphenol
SEM	Scanning Electron Microscope
XRD	X-Ray Diffraction
