# Supplementary material for: MXene-Based High-Performance Soft Pressure Sensor Using Gel–Deep Eutectic Solvent Composite
Source: Micromachines (Basel). 2025 May 15;16(5):579. doi: 10.3390/mi16050579 (PMC12114184; doi:10.3390/mi16050579)
Supplement: Supplementary file 1 [file micromachines-16-00579-s001.zip › micromachines-3563100-supplementary.pdf]

Supplementary Materials

MXene-based High-Performance Soft Pressure Sensor Using Gel-Deep Eutectic Solvent Composite

Riku Sasaki, Kaiin Tou, Shoma Kamanoi, Junya Yoshida, Yoshihiro Takabe, Yasunori Miura, Eri Kamiya, Ayana Hirayama, Tomohito Sekine*

Graduate School of Organic Materials Science, Yamagata University, 4-3-16, Jonan, Yonezawa, Yamagata 992-8510, Japan
*Correspondence: tomohito@yz.yamagata-u.ac.jp

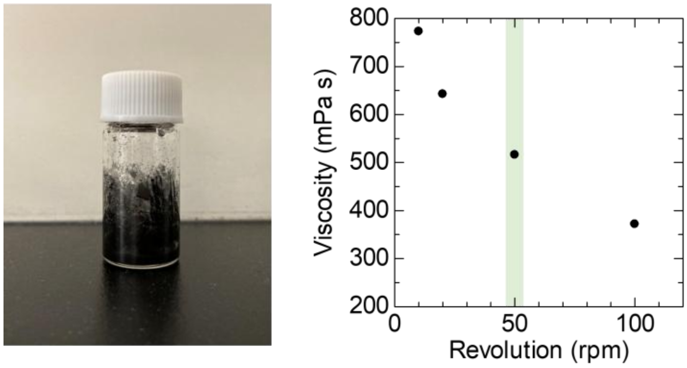

Figure S1. Viscosity measurement of composite ink using a viscometer. At a rotor speed of 50 rpm, the viscosity was approximately 515 mPa·s. The measurement was conducted using a digital viscometer (DV2T).

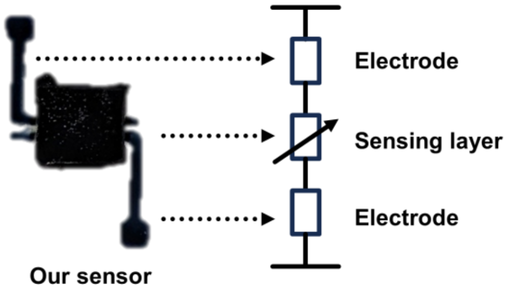

Figure S2 The equivalent circuit model of our sensor.

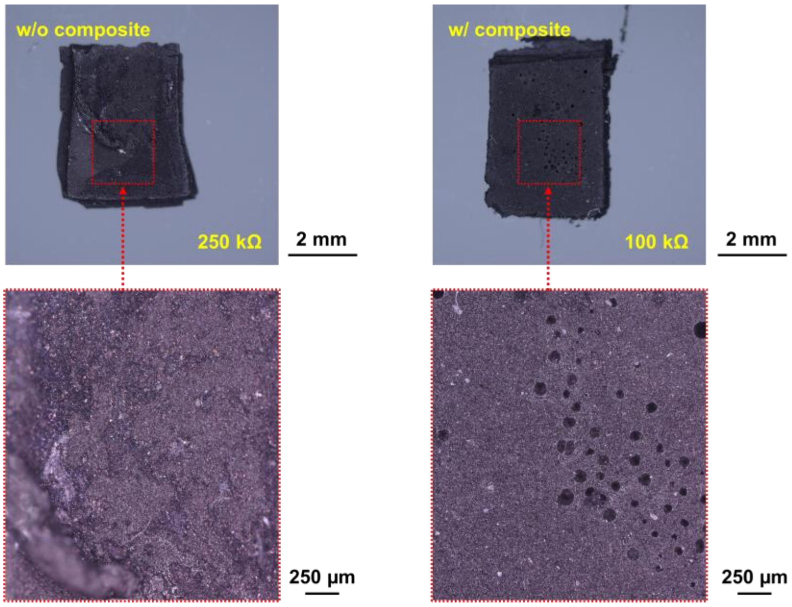

Figure S3 Morphological analysis of the pressure-sensitive layer surface using an optical microscope. The inset numbers are the initial resistances.

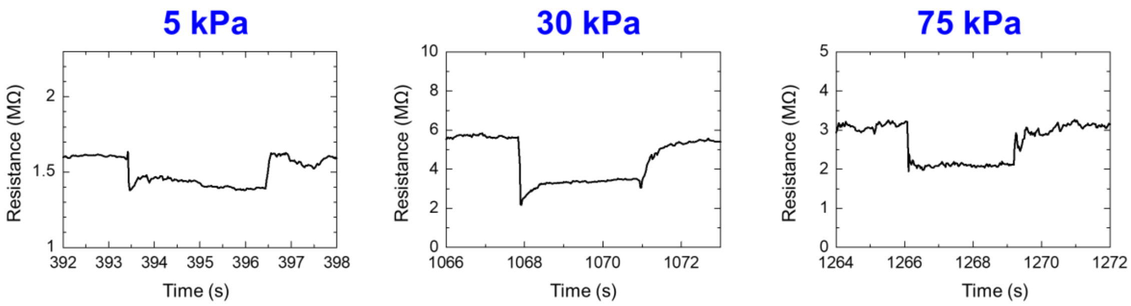

Figure S4 Representative raw data used to construct Figure 4d in the main manuscript.

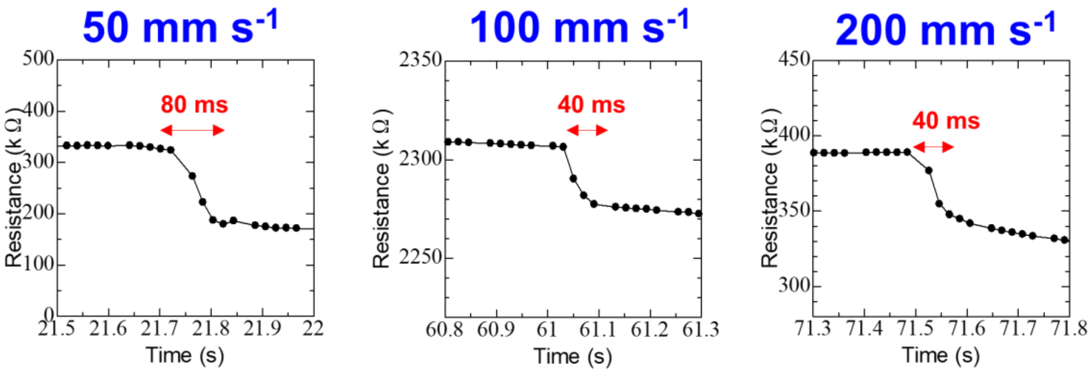

Figure S5 Response time when pressure is applied at different speeds.

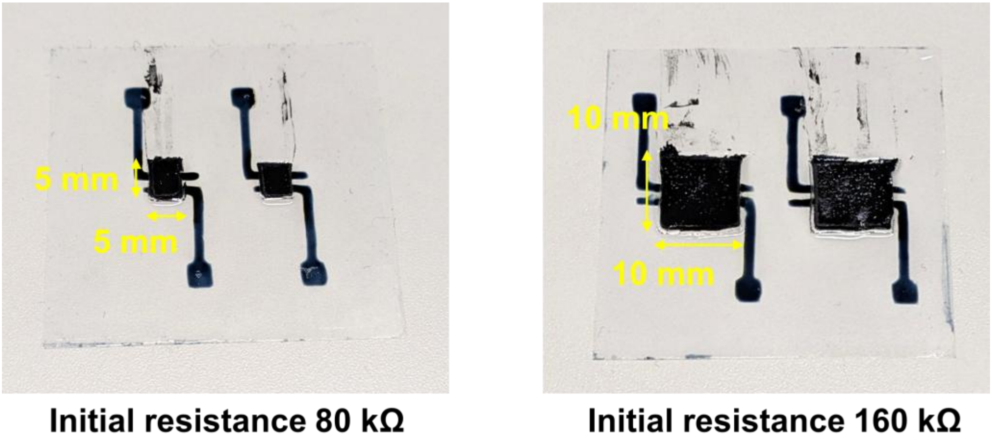

Figure S6 Relationship between the sensing layer sizes and initial resistances.

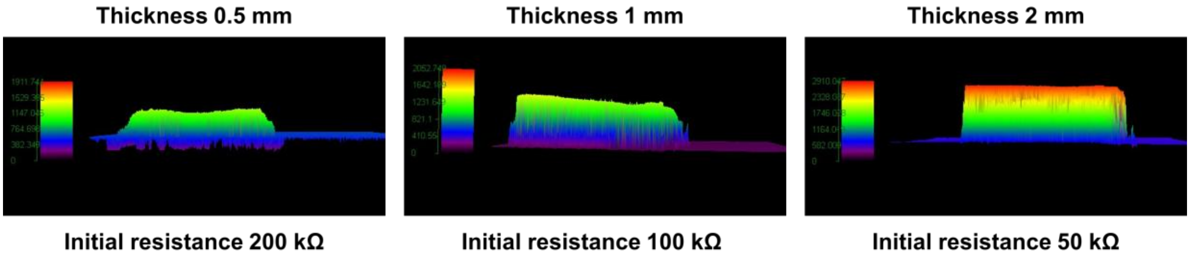

Figure S7 Relationship between sensing layer thicknesses and initial resistances.

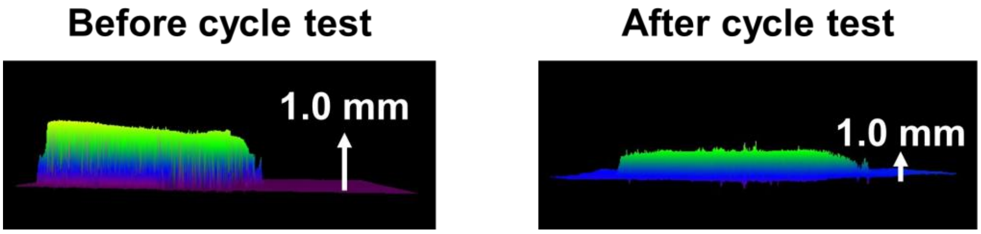

Figure S8 Cross-sectional images of the pressure-sensitive layer after 5000 testing cycles.

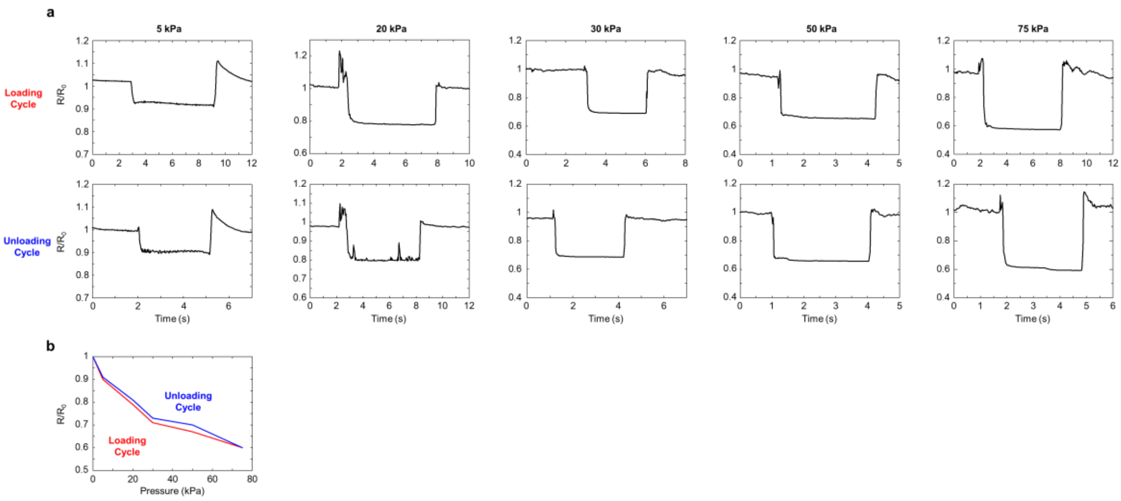

Figure S9 (a) Signal waveform used to obtain the hysteresis graph. The stress value was calculated based on the baseline and the maximum range of variation. (b) Hysteresis in the electrical characteristics of the fabricated sensor. The maximum difference in characteristics between the loading and unloading cycles was approximately 3%.

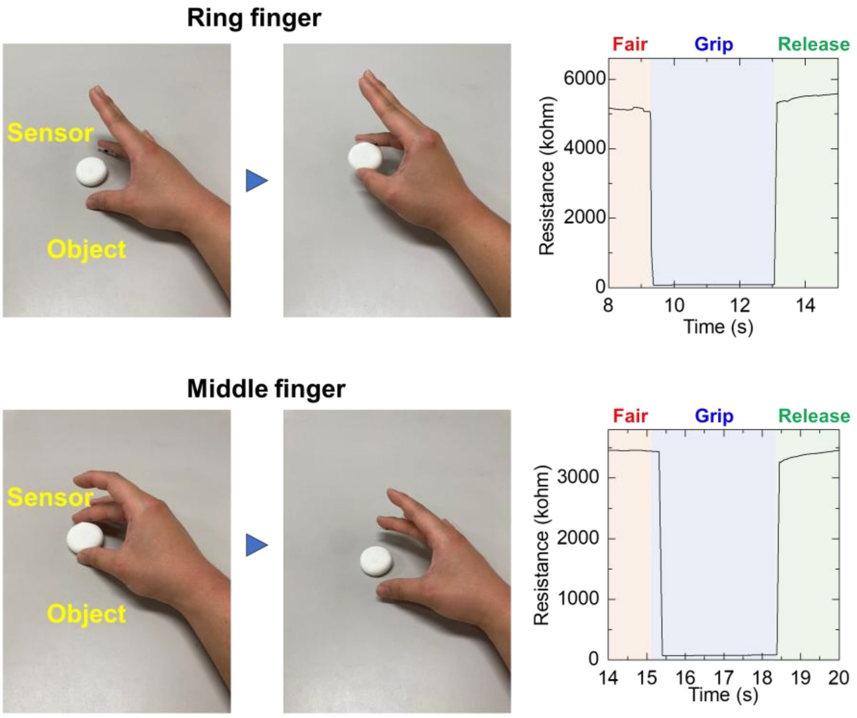

Figure S10 Additional results from the real-time grasp test conducted with the wearable device, utilizing different fingers.

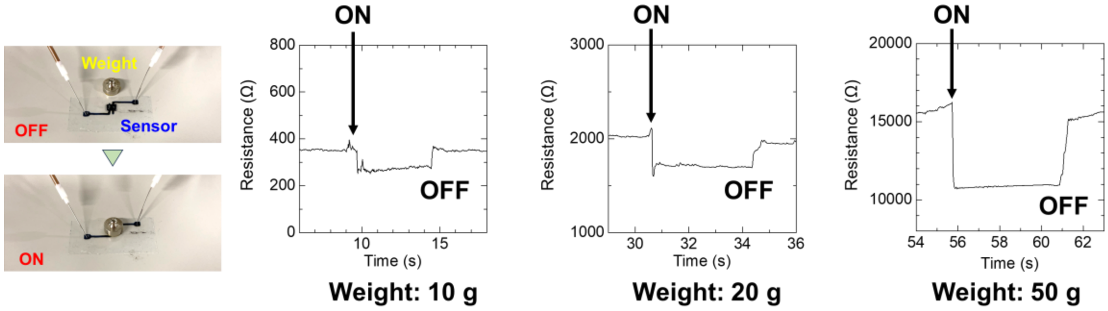

Figure S11 Vertical-pressure test for our sensor using weights of 10, 20, and 50 g.
